# A case of large gastric polyp as an adenoid cystic carcinoma metastasis

**DOI:** 10.1093/jcag/gwae019

**Published:** 2024-06-19

**Authors:** Kosuke Suzuki, Hiroaki Saito, Kimihiro Igarashi

**Affiliations:** Department of Gastroenterology, Sendai Kousei Hospital, Sendai, Miyagi, Japan; Department of Gastroenterology, Sendai Kousei Hospital, Sendai, Miyagi, Japan; Department of Internal Medicine, Soma Central Hospital, Soma, Fukushima, Japan; Department of Gastroenterology, Sendai Kousei Hospital, Sendai, Miyagi, Japan

An asymptomatic 85-year-old man was referred for treatment of a growing gastric tumour. Seven years previously, he underwent left submandibular adenectomy for adenoid cystic carcinoma (ACC) of the left submandibular gland and experienced recurrent lung metastases. Esophagogastroduodenoscopy revealed a dark reddish stalked polyp measuring 50 mm in the greater curvature of the middle gastric body (Figure 1).

**Figure 1.**
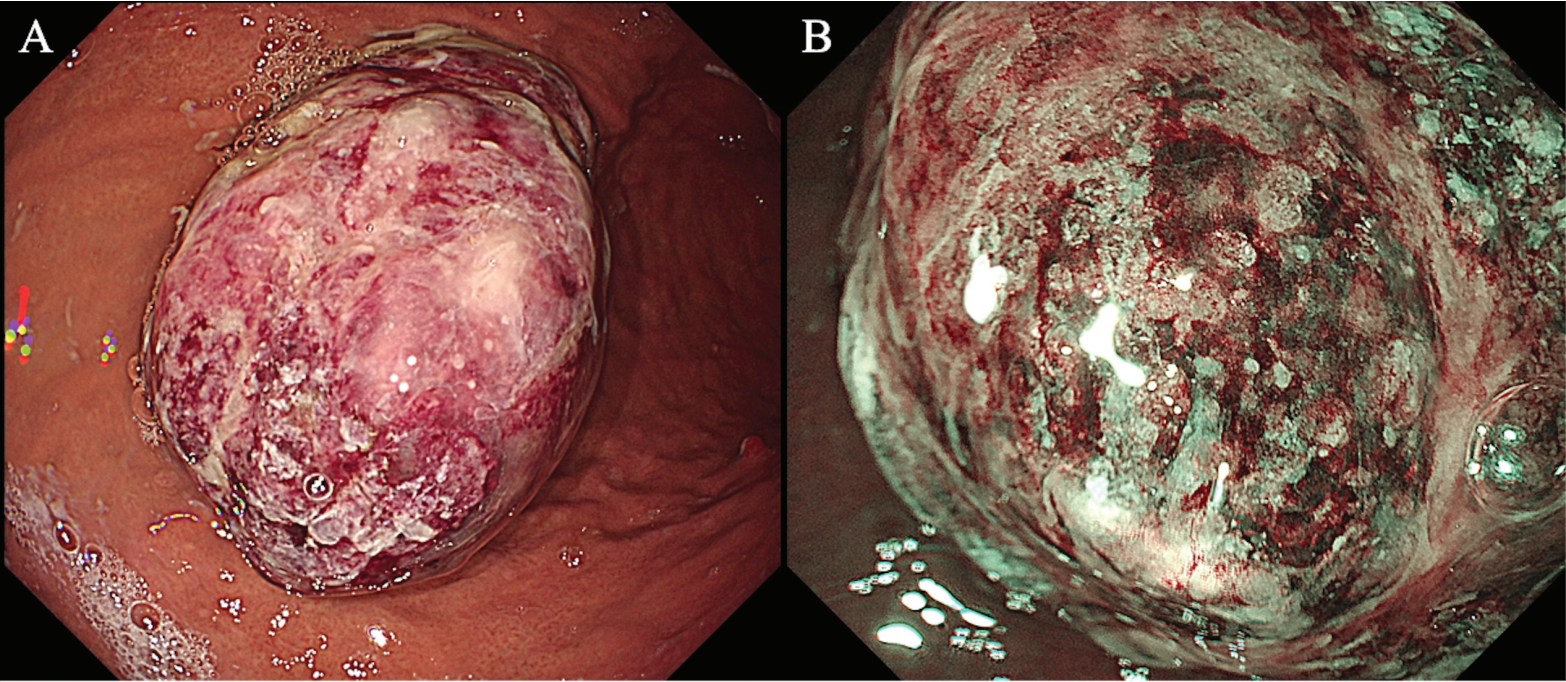
(A) White light exam. (B) Narrow band imaging exam.

We suspected the possibility of hyperplastic polyps complicated by cancer and performed an en-bloc endoscopic mucosal resection of the polyp as a total biopsy. Histopathology revealed atypical cells with various morphologies, including papillary, luminal, ribbon-like, and solid types (Figure 2A and B). Immunohistochemical results were positive for cytokeratin AE1/AE3 and negative for CDX2 and synaptophysin. The pathological findings were identical to those of the previously treated submandibular ACC (Figure 2C and D), leading to a diagnosis of gastric metastasis of the submandibular ACC. Considering the patient’s age and condition, no further anti-cancer treatments were administered. During our follow-up, no local recurrence of the stomach or the appearance of metastases in organs other than the lung was observed.

**Figure 2.**
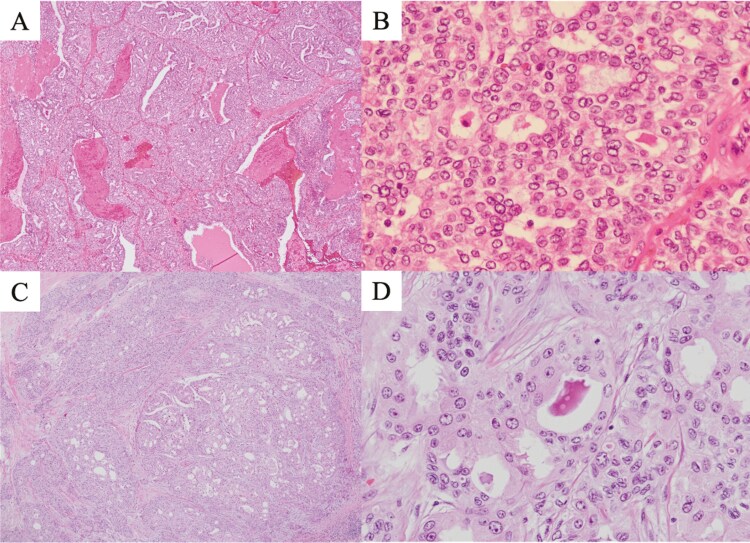
(A) The gastric specimen at low power. (B) The gastric specimen at high power. (C) The submandibular ACC specimen treated previously at low power. (D) The submandibular ACC specimen treated previously at high power.

ACC has a predilection for the salivary glands, particularly the submandibular gland and palate. Although the disease progresses slowly, distant metastases are common in the late stages. Cecal and small bowel metastases from ACC have previously been published^1^; however, few cases of gastric metastases have been reported. Diagnosing metastatic gastric tumours can be challenging as they may appear solitary or may be detected long after the primary cancer diagnosis.

## Supplementary Material

gwae019_suppl_Supplementary_Material

## Data Availability

There are no data associated with this manuscript.
